# Genomic characterization of novel viruses associated with *Olea europaea* L. in South Africa

**DOI:** 10.1007/s00705-024-06132-1

**Published:** 2024-09-27

**Authors:** David A. Read, Gerhard Pietersen, Bernard Slippers, Emma Steenkamp

**Affiliations:** 1 Department of Biochemistry, Genetics and Microbiology, Forestry and Agricultural Biotechnology Institute (FABI), University of Pretoria, Pretoria, South Africa; 2 Patho Solutions, Olifantskop Road, Wellington, 7655 South Africa

## Abstract

South Africa has a small but growing olive industry. Until now, no virological research has been carried out on this crop locally. Seventeen samples were collected from various olive cultivars from a single producer in the Stellenbosch growing area of South Africa. RNAseq was performed on total RNA, and the compositions of the metaviromes were determined. Olive leaf yellowing-associated virus was detected for the first time in South Africa, as well as four novel viruses from the family *Closteroviridae* and one each from the families *Tymoviridae* and *Solemoviridae*.

**Supplementary Information:**

The online version contains supplementary material available at 10.1007/s00705-024-06132-1.

The cultivation of olive (*Olea europaea* L.) for both table olives and oil has been an integral part of culture and agriculture in the Mediterranean basin for millennia, with orchards from Spain, Italy, and Greece spanning *ca*. 4.5 million hectares. Comparatively, South Africa’s industry has a far shorter history and comprises only *ca*. 2,400 hectares used for olive cultivation. The origins of the South African industry can be traced back to the early years of the 20th century, through the efforts of Ferdinando Costa, an agriculturalist from Genoa. Planting material was propagated from scion material imported from Italy, with native *O. europaea* subsp. *cuspidata* (wild olive) being used as rootstock material [28].

Sixteen viruses from various families have been reported in olive [1, 2]. These include olive latent virus 1, olive mild mosaic virus, tobacco necrosis virus D (*Tombusviridae*), cucumber mosaic virus, olive latent virus 2 (*Bromoviridae*), arabis mosaic virus, cherry leaf roll virus, olive latent ringspot virus, strawberry latent ringspot virus (*Secoviridae*), tobacco mosaic virus (*Virgaviridae*), olive latent virus 3 (OLV-3) (*Tymoviridae*), olive virus T (*Betaflexiviridae*), olive leaf yellowing associated virus (OLYaV) (*Closteroviridae*), olive yellow mottling and decline associated virus, and olive semilatent virus (unassigned) [1]. Another study reported that olive plants can also be infected by Olea europaea geminivirus (*Geminiviridae*) [2]. Nevertheless, when compared with other woody Mediterranean fruit crops such as grapevine and citrus, the viruses of olives are still poorly understood. Even in Spain, the largest producer of olive products, information regarding viruses infecting the crop has been described as scarce [3]. In South Africa, no previous studies have been performed on the viruses of olive.

During January 2022, samples were collected from 17 individual olive trees in commercial orchards in Stellenbosch, South Africa. Total RNA was isolated from detached petioles, using the method of White et al. [4]. RNA quality control was performed using NanoDrop (Thermo Scientific, Wilmington, DE, USA) spectrophotometry. RNAtag-seq libraries were prepared as described before [5] and sequenced using an Illumina NextSeq 2000 sequencer (Illumina, San Diego, CA, USA) at the University of Leeds NGS facility, Leeds, United Kingdom.

Sequence reads were demultiplexed into individual datasets using the Je suite [6] and trimmed for quality (quality limit = 0.05) and adapter content (Illumina universal and RNAtag-seq adapters: 5’AGATCGGAAGAG and 5’TACACGACGCTCTTCCGATCTNNNNNNNNT, respectively; see Supplementary Table S1 for the number of reads associated with each dataset, and NCBI SRA BioProject number PRJNA852409 for the trimmed sequence reads).

Each dataset is representative of the individual plant that was sampled. Reads were assembled using SPAdes 3.14.0 [7], using the *meta* option [8]. Virus and virus-like contigs were identified using BLASTn and BLASTx [9] searches against the NCBIs virus refseq database and the viral fraction of its nonredundant database, respectively. Only contigs showing similarity to plant-associated viruses were retained for further analysis. Phylogenetic analysis was performed using the amino acid (aa) sequences of the heat shock protein 70 homolog (HSP70) for closteroviruses and the RNA-dependent RNA polymerase gene for both tymoviruses and solemoviruses. These genes have been used in previous studies for comparison of related viruses [10–12]. Together with selected references from NCBI GenBank, the three aa datasets were aligned using Clustal Omega [13]. The alignments were then subjected to maximum-likelihood phylogenetic analysis in MEGA X [14], using the best-fit substitution models. The latter included the Le Gascuel (LG) substitution model [15] with empirical base frequencies (F), a proportion of invariant sites (I), and gamma distribution to account for among-site rate variation (G) for the closteroviruses, and for the sobemoviruses, the Whelan and Goldman (WAG) [16] model with G, I, and F, and for the tymoviruses, the rtREV [17] model with G, I, and F. Branch support was estimated using 1000 bootstrap replicates and the same model parameters. The positions of open reading frames were predicted using NCBI open reading frame (ORF) finder [18]. Average amino acid identity (AAI) values were determined using the Enveomics AAI tool [19].

The presence of each virus was confirmed using a two-step RT-PCR assay with M-MuLV reverse transcriptase and OneTaq 2X Master Mix (New England Biolabs, Ipswich, MA, USA) according to the manufacturer’s instructions. All resulting amplicons were sequenced in both directions by the Sanger method at the DNA Sanger sequencing facility, University of Pretoria, Pretoria, South Africa. The 5’- and 3’-terminal nucleotides of all detected viruses except for OLYaV were determined using a 5’ RACE System for Rapid Amplification of cDNA Ends, version 2.0 (Invitrogen, Carlsbad, CA, USA) and a 3’ RACE System for Rapid Amplification of cDNA Ends (Invitrogen, Carlsbad, CA, USA), respectively. A single representative sample for each novel virus was selected for both 5’ and 3’ RACE, and amplicons were sequenced in one direction using either the GSP2 or GSP primer (see Supplementary Table S2 for details regarding the RT-PCR and RACE primer sequences and the representative samples used for RACE). Genome sequences that included RACE data were considered complete and were used as exemplar genomes for comparisons. Contigs for each respective virus were aligned with the exemplar, and sequences extending beyond the confirmed 5’ and 3’ termini of the exemplar were considered to be assembly artefacts and were trimmed. Except where noted otherwise, the contigs of all variants appeared to be complete. The variant representing the exemplar for each virus is underlined under each of the subheadings below.

Six putatively novel viruses were detected in olive tissues in this study (Supplementary Table S3, Fig. 1). Of these, four apparently belonged to three genera in the family *Closteroviridae* (*Velarivirus*, *Ampelovirus*, and *Olivavirus*) and one each belonged to the family *Tymoviridae* (genus *Marafivirus*) and the family *Solemoviridae* (genus *Sobemovirus*). The HSP70 gene product of putative closteroviruses showed > 25% amino acid sequence divergence when compared with the cognate gene product of the most closely related known virus. This suggests that, except for OLYaV, the closteroviruses found in this study are new [20]. The NCBI GenBank numbers for each of the viral genomes described in this study, together with their average coverage values, are listed in Supplementary Table S4.

**Fig. 1:**
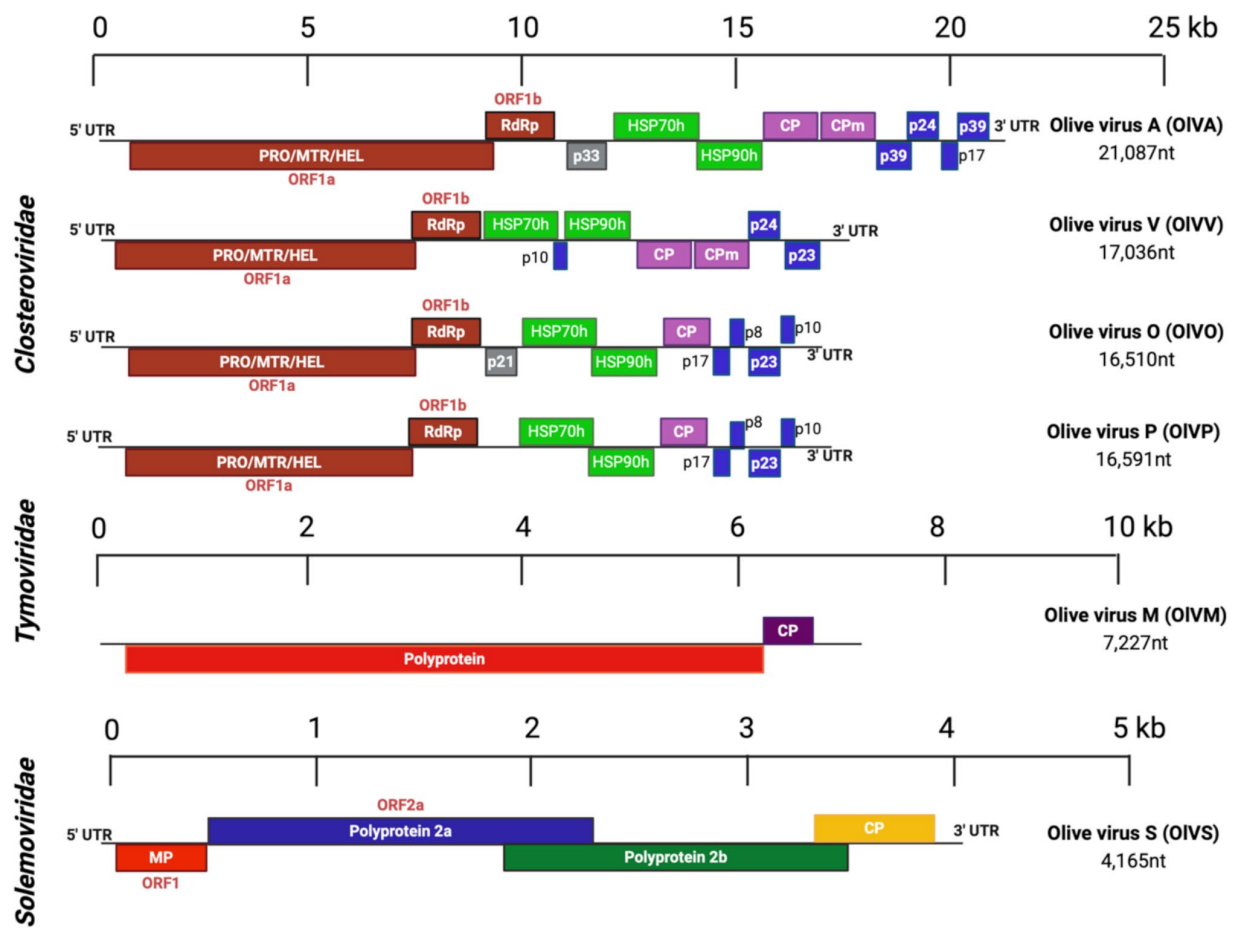
Genome organisation of novel olive-associated viruses. PRO, protease; MTR, methyltransferase; HEL, helicase; RdRP, RNA-dependent RNA polymerase; HSP70h, heat shock protein 70 homolog; HSP90h, heat shock protein 90 homolog; CP, coat protein; CPm, minor coat protein; MP, movement protein

## Velarivirus

**Fig. 2:**
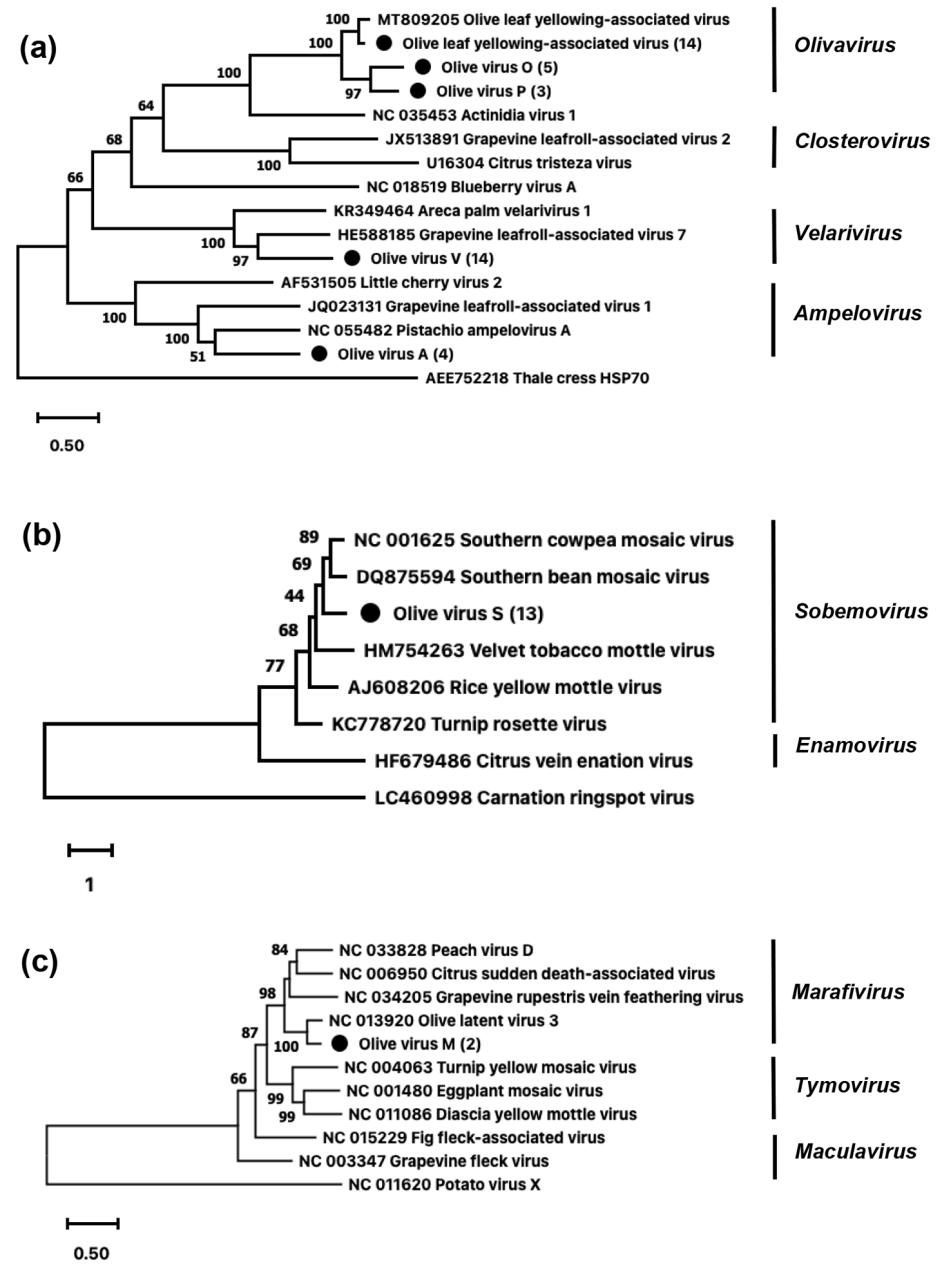
Abridged maximum likelihood trees illustrating the phylogenetic relationships between the novel (indicated by circles) (**a**) closteroviruses (heat shock protein 70 homolog), (**b**) sobemovirus (RNA-dependent RNA polymerase (RdRp), ORF2a) and (**c**) marafivirus (RdRp) with selected references from GenBank. The number of variants representing each virus from this study is shown in brackets

A novel velarivirus, tentatively named "olive virus V" (OlVV), was associated with metaviromes derived from four cultivars, namely Coratina, Mission, Frantoio, and Kalamata, representing 14 metaviromes (22–0038, 22–0039, 22–0040, 22–0041, 22–0042, 22–0043, 22–0044, 22–0046, 22–0047, 22–0049, 22–0051, 22–0052, 22–0053, and 22–0054. This virus has a genome length between 17,035 and 17,036 nt with a modal length of 17,035 nt. The OlVV genome sequence obtained from sample 22–0054 appears to be incomplete, with a length of 16,173 nt. Putative gene products share between 22–63% AAI with cordyline virus 1, grapevine leafroll-associated virus 7 (GLRaV-7), and agapanthus velarivirus (AgVV) (Supplementary Table S3), and this was supported by the closterovirus HSP70 phylogeny (Fig. 2; Supplementary Fig. S1). The population of OlVV in the samples appears to be highly homogenous, with > 99.7% nucleotide sequence identity for all variants. The genome organisation appears to be most like that of GLRaV-7 [21]. The predicted products, denoted as p10, p26, and p23, did not share significant sequence similarity with any currently known proteins in the databases. Generally, the capacity of velariviruses to induce disease symptoms has been difficult to quantify. For example, cordyline viruses have been associated with both disease and seemingly latent infections [22]. On the other hand, little cherry virus 1 has been consistently associated with little cherry disease, although this appears to be in synergism with another closterovirus, little cherry virus 2 [23].

## Ampelo-like virus

Four metaviromes derived from samples 22–0040, 22–0046, 22–0047, and 22–0052 contained a novel virus with features similar to those of members of the genus *Ampelovirus* (Supplementary Table S3). This virus has been provisionally named "olive virus A" (OlVA). The genome lengths vary between 21,057 (two variants) and 21,087 nt (two variants). The highest amino acid sequence similarity was found with grapevine leafroll associated virus 1, pistachio ampelovirus A (PAVA), citrus associated ampelovirus, and citrus associated ampelovirus 2, with 23–57% identity. This is supported by the phylogeny of the HSP70 gene (Fig. 2, Supplementary Fig. S1). Average nucleotide identity (ANI) values varied between 92 and 99.8% between OlVA genomes. The phylogeny (Fig. 2; Supplementary Fig. S1) shows that OlVA groups most closely with PAVA. Variants of citrus tristeza virus have long been considered to have the largest unsegmented plant RNA genomes, at 19,302 nt. The genome of OlVA exceeds this by at least 1,755nt.

In addition to its exceptional genome length, OlVA is also remarkable in terms of sequence divergence and the presence of at least four putative genes with no known homologs. These characteristics seem to point to a highly divergent lineage within the family *Closteroviridae*. However, the homology of the genes from the replicase through to the capsid proteins to other extant ampeloviruses suggests that OlVA belongs to the genus *Ampelovirus*. When considering the genes beyond those of the capsid proteins, closteroviruses show a staggering variation in number, function, and origin, with little to no similarity to known proteins [24].

## Olivaviruses

Two viruses that appear to belong to the genus *Olivavirus* were discovered in this study (Supplementary Table S3). These viruses have been tentatively named "olive virus O" (OlVO) and "olive virus P" (OlVP) and were associated with five samples from the Coratina (22–0038, 22–0039, 22–0050, 22–0051) and Frantoio (22–0054) cultivars and three samples from the Mission (22–0040, 22–0052, 22–0053) cultivar. The complete genome lengths of variants of OlVO ranged between 16,507 and 16,512 nt. Populations of OlVO appear to be variable, with the ANI between variants between 86.6% and 99.9%. Putative gene products share 46.1–73.6% AAI with variants of olive leaf yellowing-associated virus (OLYaV). The complete genome length of OlVP is 16,590 nt, and the ANI between variants is between 99.6% and 99.8%. OLYaV was first associated with leaf yellowing symptoms of olive in Italy [25]. Plants associated with OlVO and OlVP showed variable virus-like symptoms. The latter included 10 samples (22–0038, 22–0040, 22–0041, 22–0042, 22–0043, 22–0045, 22–0048, 22–0049, 22–0050, 22–0052) infected with one to four variants of OLYaV, which is also a member of the genus *Olivavirus*. Also, OLYaV variants from this study shared ANI values of between 71 and 100% with previously characterized variants from Brazil, Spain, and Greece.

## Marafivirus

The provisionally named "olive virus M" (OlVM) is a putative member of the genus *Marafivirus.* It was associated with samples 22–0047 and 22–0051, collected from the Frantoio and Coratina cultivars, respectively. OlVM shares the greatest sequence similarity with olive latent virus 3 (OLV-3) but falls below the species demarcation thresholds [26] for members of the genus, sharing 80% AAI for the capsid protein and 67.3% ANI between the genomes of the two viruses. OlVM has a genome length of 7,223-7,224 nt, encoding two putative gene products (Supplementary Table S3): a replicase and a capsid protein. The relationship to OLV-3 was confirmed by phylogenetic analysis based on the replicase gene product (Fig. 2, Supplementary Fig. S3). The two variants of OlVM shared 98% ANI with each other. OlVM was the virus with the lowest incidence in this study.

## Sobemovirus

The presence of a putative member of the family *Solemoviridae* was determined in 10 samples (22–0038, 22–0040, 22–0043, 22–0044, 22–0045, 22–0046, 22–0049, 22–0051, 22–0052, and 22–0053). The proposed name of the virus is "olive virus S" (OlVS). Genome lengths varied between 4,157 and 4165 nt. Nucleotide sequence variability between variants ranged from 96.9 to 99.5%. OlVS shares the most sequence similarity with southern bean mosaic virus (SBMV), with an average ANI of 52.5%. This value is below the sequence-based species demarcation guideline of ∼ 75% complete genome ANI [27]. This relationship was confirmed through phylogenetic analysis (Fig. 2, Supplementary Fig. S2). The OlVS genome contains four putative genes (Supplementary Table S3). The putative ORF1 product showed no homology to known proteins, but it is presumed to be a movement protein. The ORF 2–4 products show 39.5–50.4% AAI to proteins encoded by other sobemoviruses. While there are currently no prescribed sequence-identity-based genus delineation thresholds, the phylogenetic grouping of OlVS with sobemoviruses suggests that it is a putative member of the genus *Sobemovirus*.

The current study adds to the growing body of information on viruses of olive. No virus studies have been carried out previously on olive in South Africa, and even in countries with large-scale olive production, knowledge about the diversity and distribution of olive viruses is scant. The use of non-targeting high-throughput sequencing (HTS) has only recently been applied for studying virus diversity in this crop [1–3, 29]. The apparent lag in virus research on olive, relative to other major fruit crops, is likely due to latent infectons apparently caused by most olive-associated viruses found so far [3]. Viral latency is a polysemous term, but in plant virology, it generally refers to a viral infection that is not associated with any visible symptoms [30]. Foliar symptoms are often the most “clear”, with other less obvious symptoms such as effects on yield and metabolite production being more diffcult to observe and quantify. The tough, leathery texture of the olive leaf might play a role in the apparent latency of foliar symptoms and the concomitant lag in virological research.

The discovery of the four novel closteroviruses in this study, from a single host species at a single location, is extraordinary. In fact, of the seven viruses that were discovered during this study, only OLYaV had been characterised previously. In a recent study on the viral diversity of wild citrus hosts, the presence of three novel closteroviruses from two putative genera was considered remarkable [31]. The authors argued that the closterovirus diversity observed on wild citrus (and absent from cultivated varieties) may be due to the physical isolation of the hosts. While information on the origins of olive cultivars examined in the current study remains unclear and a source of debate [32], cultivated olives are inextricably linked to the Mediterranean basin and the wild oleaster (*O. europaea* subsp. *sylvestris*). Unlike the case of citrus, range overlap and hybridisation between olive and oleaster have probably been occurring for millennia [32]. It is therefore reasonable to hypothesise that olive and oleaster share many of the same viruses, at least in the Mediterranean basin. However, except for OLYaV, all of the viruses from this study are novel and were not described previously in any other location. This raises the very pertinent question of the origin of these viruses. While further research is required to answer this question, the apparent absence of these viruses in olive elsewhere in the world suggests that they may have originated from a source native to South Africa. A plausible hypothesis would be that these viruses originated in wild olive, especially since it was used as a source of rootstock material during the establishment of the industry, placing it in direct contact with scions along with any associated graft-transmissible entities. Investigating the possible route of transmission is an area of active and ongoing research.

The pathology of these newly discovered viruses should be determined. Many of the known closteroviruses infect fruit trees [20] and can lead to significant economic losses [33]. In addition to the discovery of novel viruses, this study has shed significant light on the diversity of OLYaV, which, despite being discovered in 1999 [25], was only recently genomically characterised [29]. In fact, this study accounts for the greatest amount of complete genomic data for OLYaV to date. Despite the number and diversity of closterovirids discovered in this study, OLYaV is the only one reported previously to be associated with olive. Fontana et al. [34] determined that OLYaV has “no negative interference” with oil quality and yield. However, the numbers of plants analysed in that study was limited. and OLYaV infections have been associated with woody cylinder deformations [3], a symptom often associated with closterovirid infections. Further research into the potential negative effects of OLYaV infections are therefore warranted, particularly since OLYaV appears to be particularly widespread.

OlVM is the second member of the family *Tymoviridae* found to date in olive plants. OlVM appears to have only two ORFs, whereas OLV-3 has four ORFs. Tymoviruses of the same genera can often have different numbers of putative ORFs [26]. As well as sharing sequence similarity with OLV-3, OlVM shares other physicochemical attributes, such as a genome length of ∼ 7,200 nt and a coat protein molecular mass of ∼ 29 kDa [35]. Given these shared characteristics, it is plausible that OlVM and OLV3 belong to the same genus of the family *Tymoviridae*.

Olive latent virus 1 (OLV-1) was previously considered to be a member of the genus *Sobemovirus* [36, 37] but was later assigned to the genus *Necrovirus* (now *Alphanecrovirus*) of the family *Tombusviridae* [38]. Based on sequence homology and the phylogenetic results, OlVS is most likely a member of the genus *Sobemovirus*, making it the first member of this taxon (and the family *Solemoviridae*) to be associated with olive. Sobemoviruses are mainly transmitted through mechanical wounding [39] and vegetative propagation. The presence of a sobemovirus associated with olive is in itself curious, since sobemoviruses appear to have deep evolutionary associations with the families Poaceae, Fabaceae, and Solanaceae [38]. The genus *Sobemovirus* includes many economically important viruses; however, some of its members have been found in latent infections [40]. The effect of the association of OlVS with olive remains unknown.

In conclusion, seven viruses, six of which are novel, were detected in 17 olive trees in the Stellenbosch region of South Africa. Our study thus represents a significant advancement in our understanding of olive virology at both a regional and global level. Most newly discovered viruses are associated with a paucity of biological data, and the viruses in this study are no exception. However, this work has laid the foundation for ongoing research on the diversity, distribution, and the potential origin of these viruses.

## Electronic Supplementary Material

Below is the link to the electronic supplementary material

Supplementary Material 1

Supplementary Material 2

Supplementary Material 3

Supplementary Material 4

Supplementary Material 5

Supplementary Material 6

Supplementary Material 7

## Data Availability

The data that support the findings of this study are openly available in NCBI public databases.
