# Supplementary material for: Genomic characterization of novel viruses associated with Olea europaea L. in South Africa
Source: Arch Virol. 2024 Sep 27;169(10):210. doi: 10.1007/s00705-024-06132-1 (PMC11427506; doi:10.1007/s00705-024-06132-1)
Supplement: Supplementary file 1 — Supplementary Material 1 [file 705_2024_6132_MOESM1_ESM.pdf]

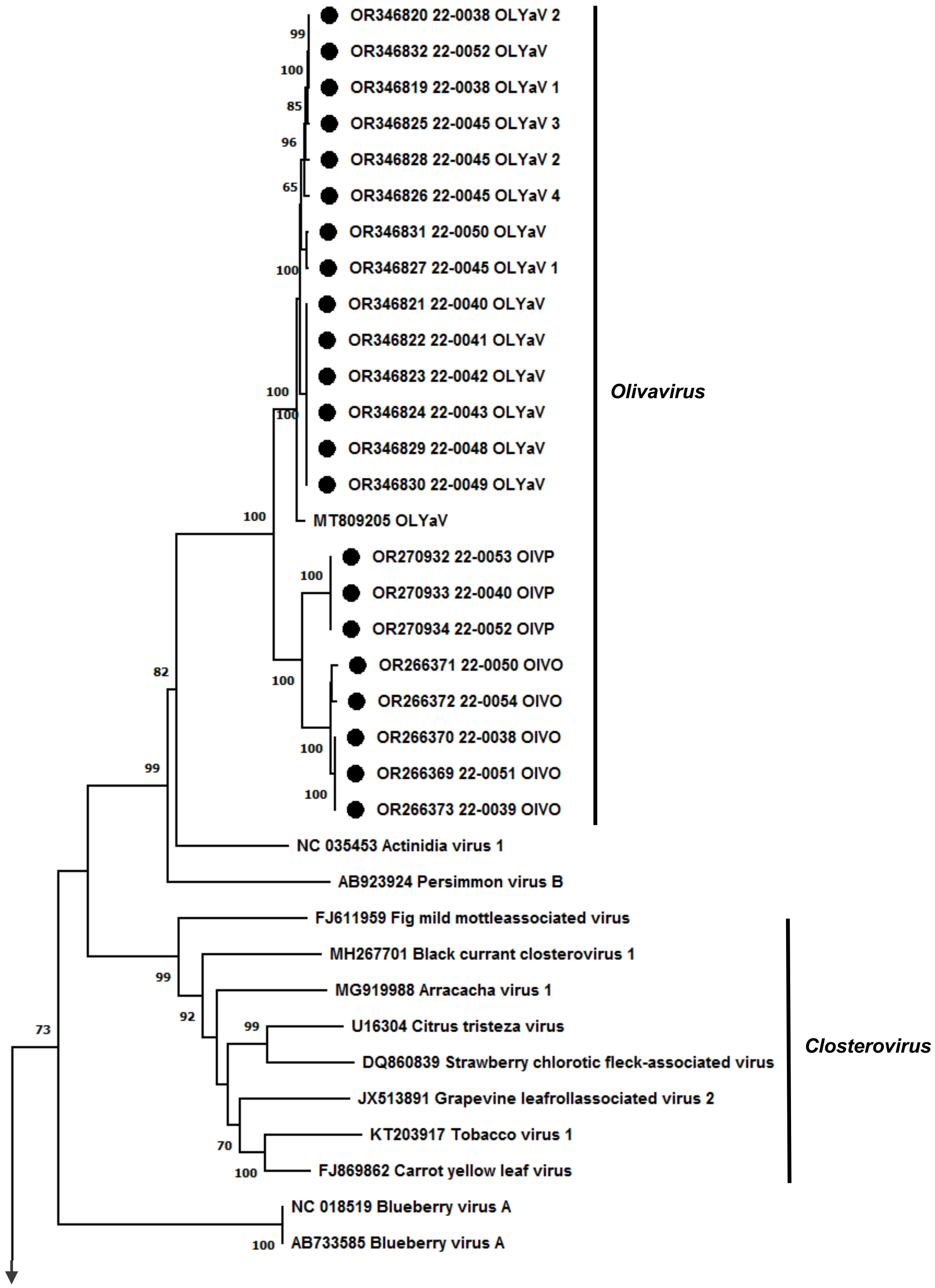

OR346820 22-0038 OLYaV 2
OR346832 22-0052 OLYaV
OR346819 22-0038 OLYaV 1
OR346825 22-0045 OLYaV 3
OR346828 22-0045 OLYaV 2
OR346826 22-0045 OLYaV 4
OR346831 22-0050 OLYaV
OR346827 22-0045 OLYaV 1
OR346821 22-0040 OLYaV
OR346822 22-0041 OLYaV
OR346823 22-0042 OLYaV
OR346824 22-0043 OLYaV
OR346829 22-0048 OLYaV
OR346830 22-0049 OLYaV
MT809205 OLYaV
OR270932 22-0053 OlVP
OR270933 22-0040 OlVP
OR270934 22-0052 OlVP
OR266371 22-0050 OlVO
OR266372 22-0054 OlVO
OR266370 22-0038 OlVO
OR266369 22-0051 OlVO
OR266373 22-0039 OlVO
Olivavirus
NC 035453 Actinidia virus 1
AB923924 Persimmon virus B
FJ611959 Fig mild mottleassociated virus
MH267701 Black currant closterovirus 1
MG919988 Arracacha virus 1
U16304 Citrus tristeza virus
DQ860839 Strawberry chlorotic fleck-associated virus
JX513891 Grapevine leafrollassociated virus 2
KT203917 Tobacco virus 1
FJ869862 Carrot yellow leaf virus
Closterovirus
NC 018519 Blueberry virus A
AB733585 Blueberry virus A
99
100
85
96
65
100
100
100
100
82
100
100
100
100
99
99
92
99
70
100
73
100

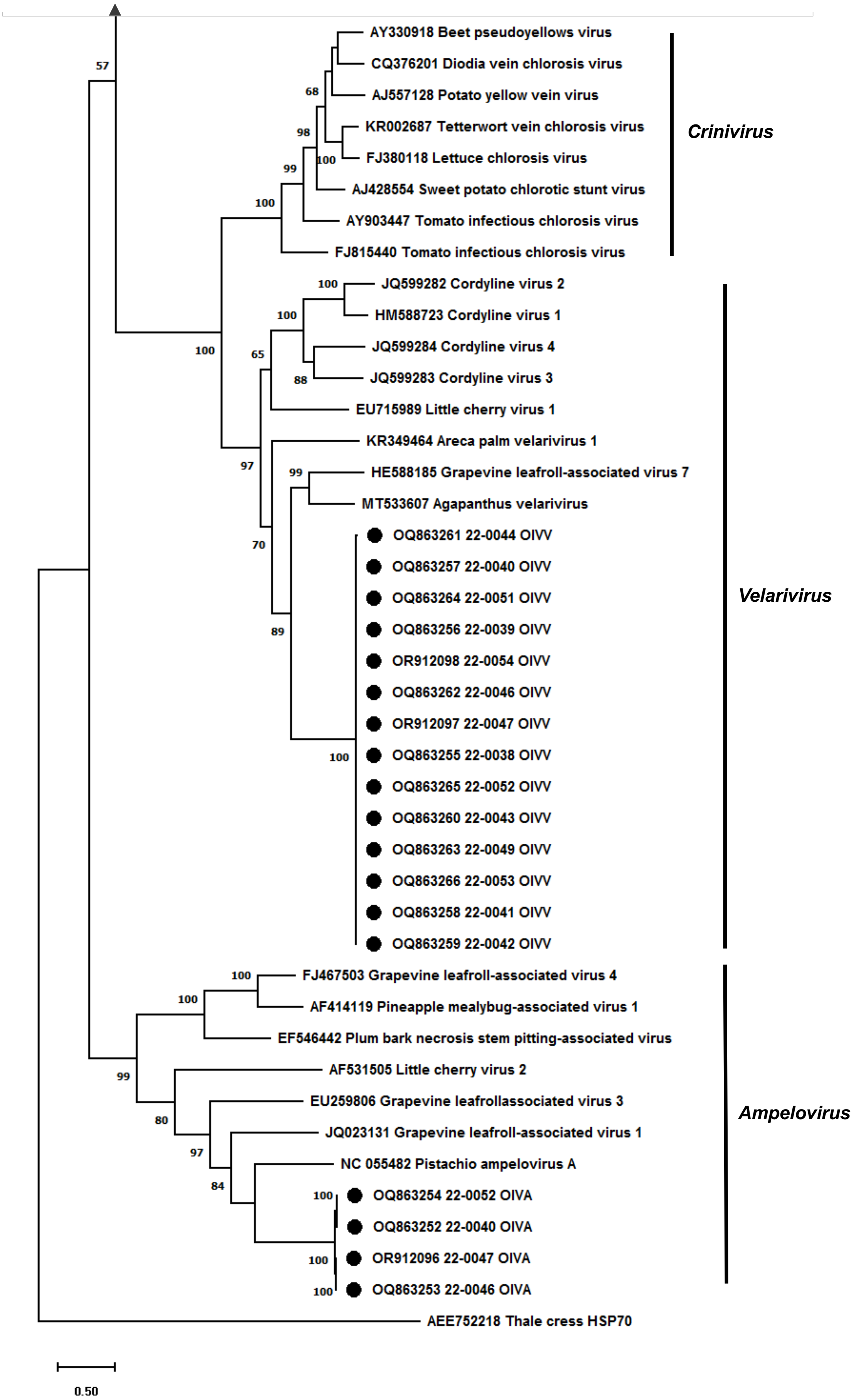

Supplementary Figure S1: Maximum likelihood phylogeny based on the amino acid sequences of the heat shock protein 70 homolog (HSP70h) of closteroviruses from this study (variants from this study indicated by solid circle markers) and selected members of the *Closteroviridae* family. The phylogeny represents the tree with the highest log likelihood and was generated in *MEGA X* using the Le Gascuel model (Le Gascuel, 2008) with empirical base frequencies, a proportion of invariant sites and gamma distribution to account for among-site rate variation. Bootstrapping was applied (1000 replicates) and the percentage of trees in which the associated taxa clustered together is shown next to the branches. Bootstrap percentages lower than 50 are not shown. A HSP70h amino acid sequence of *Arabidopsis thaliana* was used as an outgroup.
