# Supplementary material for: Genomic characterization of novel viruses associated with Olea europaea L. in South Africa
Source: Arch Virol. 2024 Sep 27;169(10):210. doi: 10.1007/s00705-024-06132-1 (PMC11427506; doi:10.1007/s00705-024-06132-1)
Supplement: Supplementary file 2 — Supplementary Material 2 [file 705_2024_6132_MOESM2_ESM.pdf]

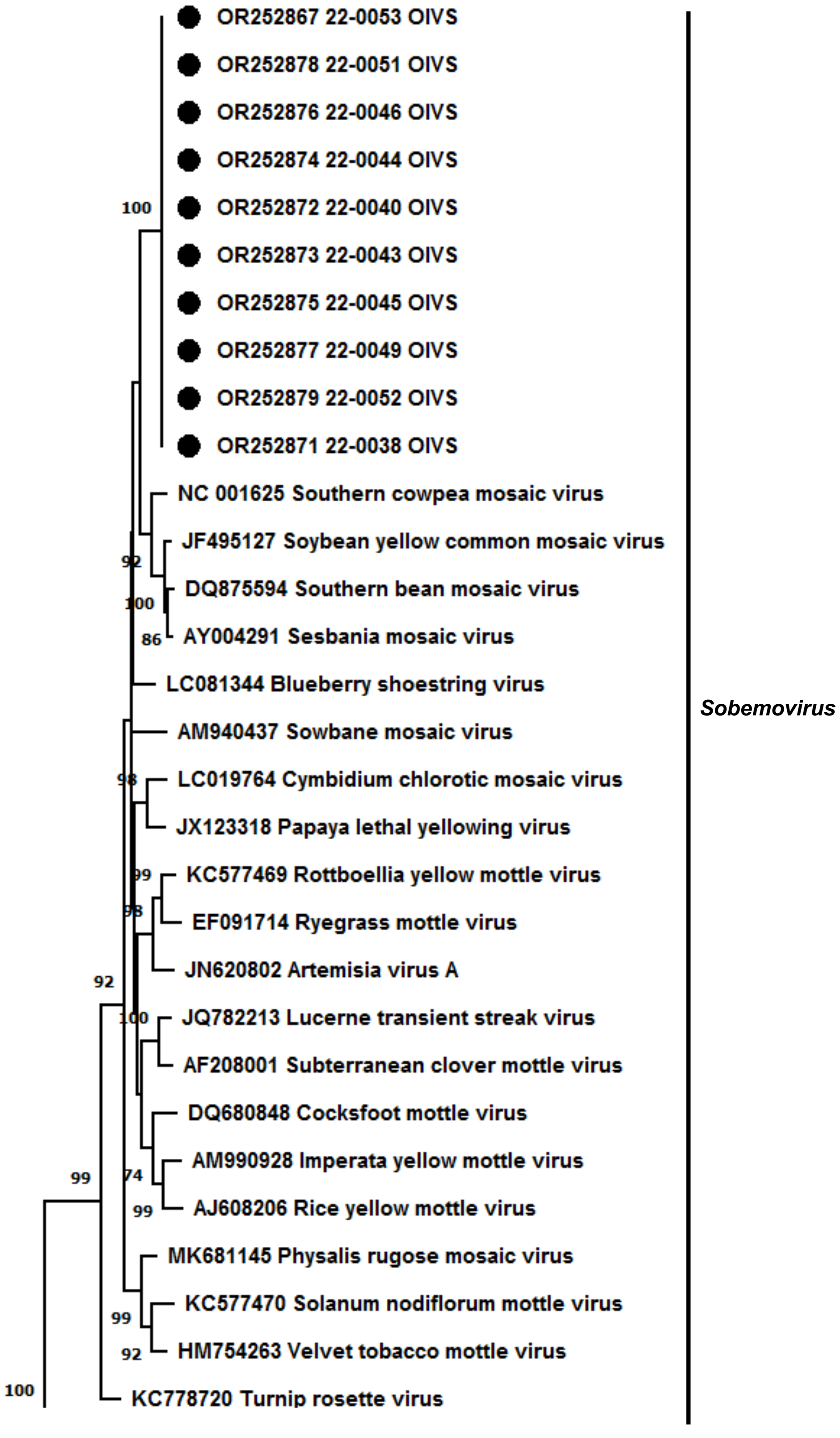

OR252867 22-0053 OIVS
OR252878 22-0051 OIVS
OR252876 22-0046 OIVS
OR252874 22-0044 OIVS
OR252872 22-0040 OIVS
OR252873 22-0043 OIVS
OR252875 22-0045 OIVS
OR252877 22-0049 OIVS
OR252879 22-0052 OIVS
OR252871 22-0038 OIVS
NC 001625 Southern cowpea mosaic virus
JF495127 Soybean yellow common mosaic virus
DQ875594 Southern bean mosaic virus
AY004291 Sesbania mosaic virus
LC081344 Blueberry shoestring virus
AM940437 Sowbane mosaic virus
LC019764 Cymbidium chlorotic mosaic virus
JX123318 Papaya lethal yellowing virus
KC577469 Rottboellia yellow mottle virus
EF091714 Ryegrass mottle virus
JN620802 Artemisia virus A
JQ782213 Lucerne transient streak virus
AF208001 Subterranean clover mottle virus
DQ680848 Cocksfoot mottle virus
AM990928 Imperata yellow mottle virus
AJ608206 Rice yellow mottle virus
MK681145 Physalis rugose mosaic virus
KC577470 Solanum nodiflorum mottle virus
HM754263 Velvet tobacco mottle virus
KC778720 Turnip rosette virus
Sobemovirus
100
92
100
86
98
99
98
92
100
74
99
99
99
92
100

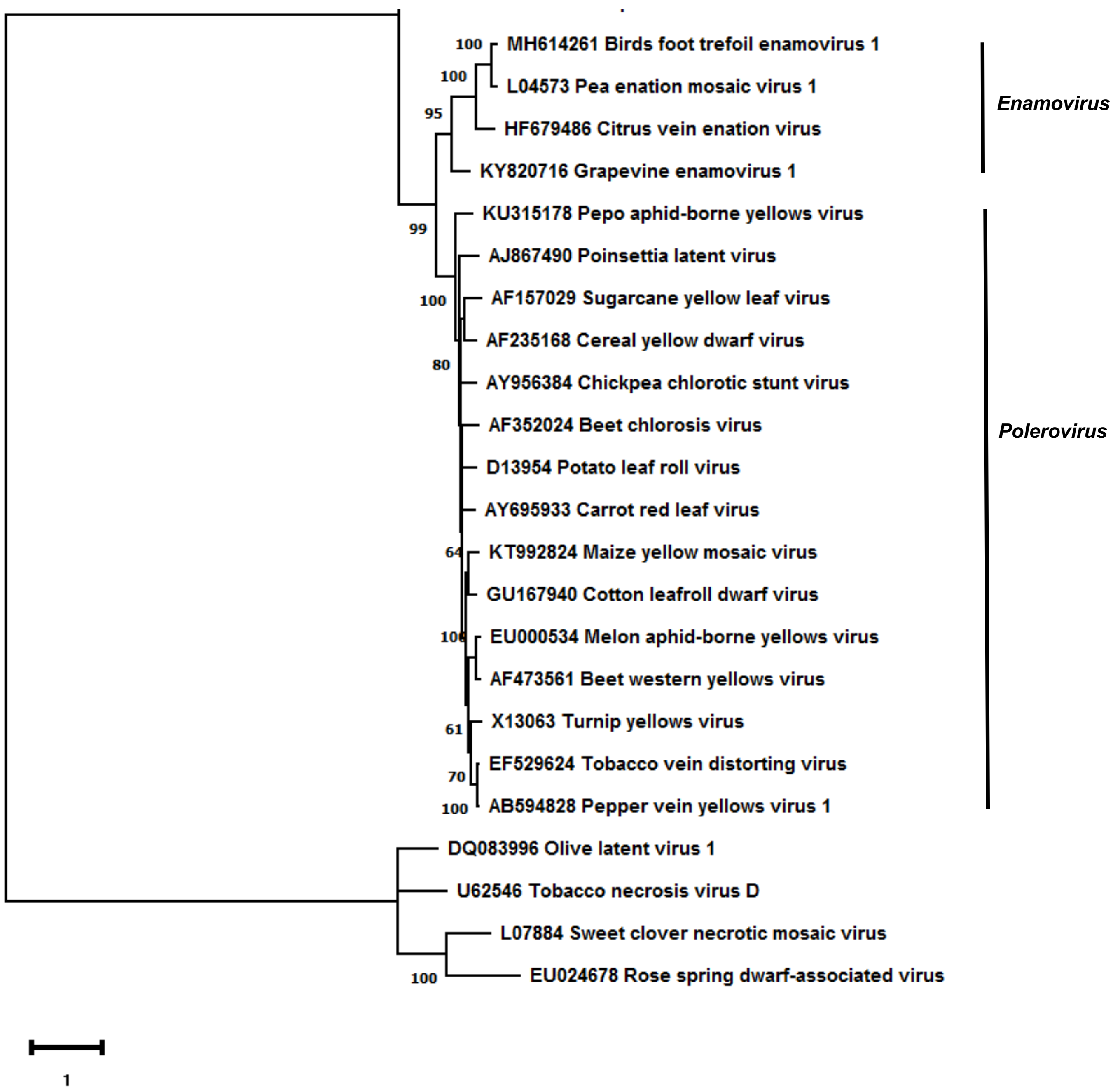

Supplementary Figure S2: Maximum likelihood phylogeny based on the amino acid sequences of the RNA-dependant RNA polymerase of olive virus S from this study (variants from this study indicated by solid circle markers) and selected members of the *Solemoviridae* family. The phylogeny represents the tree with the highest log likelihood and was generated in *MEGA X* using the Whelan and Goldman (WAG) model with empirical base frequencies, a proportion of invariant sites and gamma distribution to account for among-site rate variation. Bootstrapping was applied (1000 replicates) and the percentage of trees in which the associated taxa clustered together is shown next to the branches. Bootstrap percentages lower than 50 are not shown.
