# Supplementary material for: Genomic characterization of novel viruses associated with Olea europaea L. in South Africa
Source: Arch Virol. 2024 Sep 27;169(10):210. doi: 10.1007/s00705-024-06132-1 (PMC11427506; doi:10.1007/s00705-024-06132-1)
Supplement: Supplementary file 3 — Supplementary Material 3 [file 705_2024_6132_MOESM3_ESM.pdf]

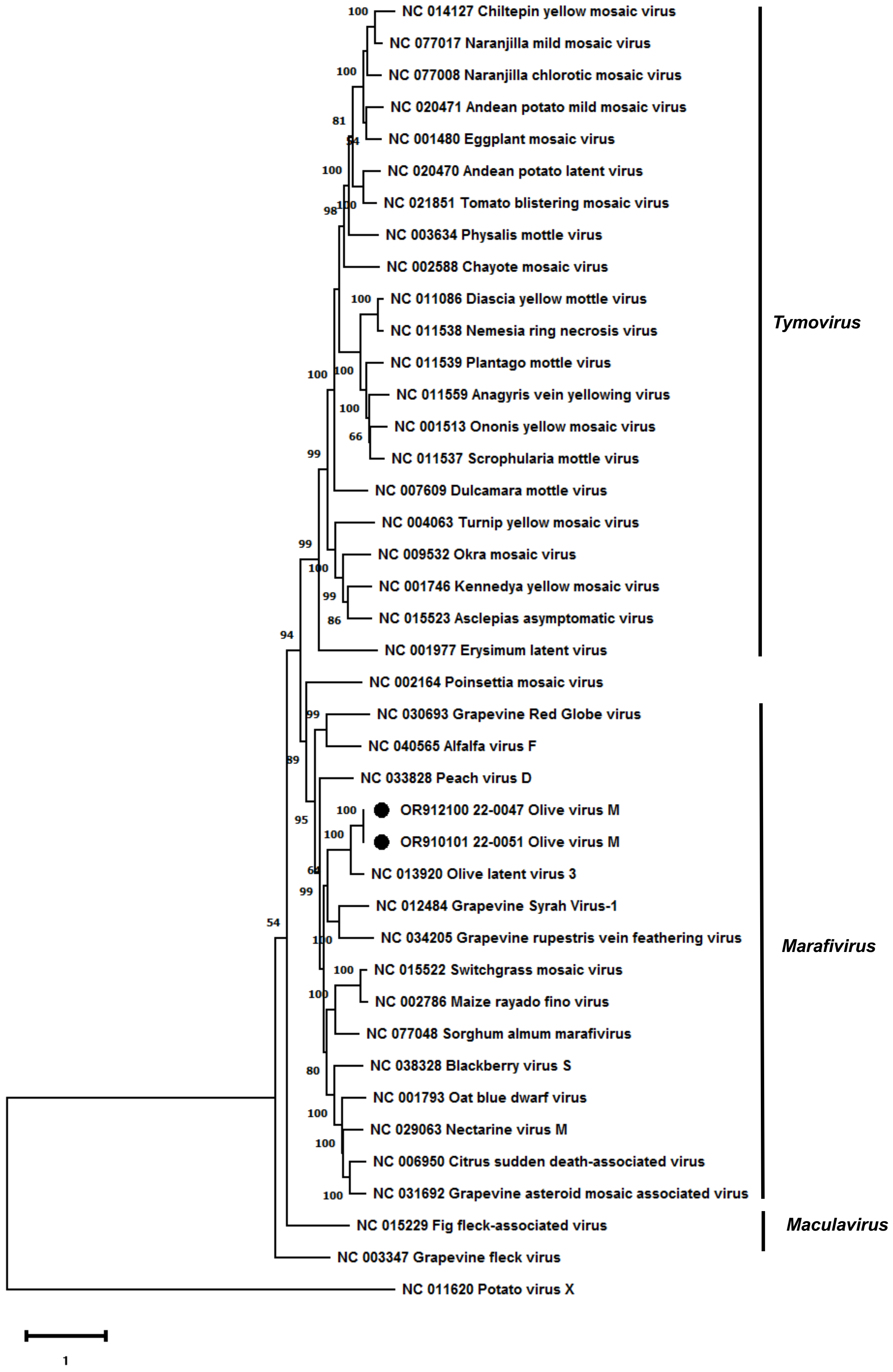

Supplementary Figure S3: Maximum likelihood phylogeny based on the amino acid sequences of the RNA-dependant RNA polymerase of olive virus M from this study (variants from this study indicated by solid circle markers) and selected members of the *Tymoviridae* family. The phylogeny represents the tree with the highest log likelihood and was generated in *MEGA X* using the rtREV model with empirical base frequencies, a proportion of invariant sites and gamma distribution to account for among-site rate variation. Bootstrapping was applied (1000 replicates) and the percentage of trees in which the associated taxa clustered together is shown next to the branches. Bootstrap percentages lower than 50 are not shown.
