# Supplementary material for: Genomic characterization of novel viruses associated with Olea europaea L. in South Africa
Source: Arch Virol. 2024 Sep 27;169(10):210. doi: 10.1007/s00705-024-06132-1 (PMC11427506; doi:10.1007/s00705-024-06132-1)
Supplement: Supplementary file 4 — Supplementary Material 4 [file 705_2024_6132_MOESM4_ESM.docx]

| **Sample accession** | **Collection date** | **Cultivar** | **Number of reads before trim** | **Number of reads after trim** | **NCBI BioSample accession number** |
| --- | --- | --- | --- | --- | --- |
| 22-0038 | Jan-22 | Coratina | 23,497,916 | 20,924,924 | SRR23728317 |
| 22-0039 | Jan-22 | Coratina | 15,638,466 | 12,182,140 | SRR23106936 |
| 22-0040 | Jan-22 | Mission | 24,934,334 | 18,958,532 | SRR23106935 |
| 22-0041 | Jan-22 | Mission | 66,012,992 | 61,455,034 | SRR23728316 |
| 22-0042 | Jan-22 | Frantoio | 53,333,922 | 46,133,442 | SRR23106934 |
| 22-0043 | Jan-22 | Kalamata | 27,469,854 | 25,619,263 | SRR23728315 |
| 22-0044 | Jan-22 | Kalamata | 11,276,794 | 6,581,519 | SRR23106933 |
| 22-0045 | Jan-22 | Frantoio | 79,925,050 | 73,604,216 | SRR23728314 |
| 22-0046 | Jan-22 | Frantoio | 15,307,068 | 14,062,168 | SRR23106932 |
| 22-0047 | Jan-22 | Frantoio | 12,828,866 | 10,559,956 | SRR23106931 |
| 22-0048 | Jan-22 | Kalamata | 37,854,618 | 33,733,621 | SRR23728313 |
| 22-0049 | Jan-22 | Kalamata | 71,525,864 | 66,905,584 | SRR23728312 |
| 22-0050 | Jan-22 | Coratina | 15,949,936 | 9,054,504 | SRR23106930 |
| 22-0051 | Jan-22 | Coratina | 81,140,026 | 65,586,382 | SRR23728311 |
| 22-0052 | Jan-22 | Mission | 27,971,338 | 24,259,368 | SRR23728310 |
| 22-0053 | Jan-22 | Mission | 14,320,742 | 9,979,830 | SRR23106929 |
| 22-0054 | Jan-22 | Frantoio | 11,486,026 | 8,767,586 | SRR23106928 |

Supplementary Table S1: The number of reads before and after trimming, associated with each sample. Each sample accession and corresponding dataset is representative of an individual plant.
