# Supplementary material for: Genomic characterization of novel viruses associated with Olea europaea L. in South Africa
Source: Arch Virol. 2024 Sep 27;169(10):210. doi: 10.1007/s00705-024-06132-1 (PMC11427506; doi:10.1007/s00705-024-06132-1)
Supplement: Supplementary file 5 — Supplementary Material 5 [file 705_2024_6132_MOESM5_ESM.docx]

| **Virus** | **Primer name** | **Sequence**  **(5’ – 3’)** | **Target**  **Region** | **Target (nt)** | **Tm °C** | **Product (bp)** |
| --- | --- | --- | --- | --- | --- | --- |
| OlVA | OlVA-HSP-F | GCTGAAGCCGAAAGGAATAAACGA | HSP70 | 12451-12474 | 59 | 975 |
|  | OlVA-HSP- | GCAAGTATCCAGAAGAACAGTCTGT |  | 13401-13425 | 58 |  |
| OlVV | OlVV-HSP-F | CATTCCACGCGGTAGTTCTTACG |  | 9104-9126 | 59 | 1458 |
|  | OlVV-HSP-R | AGGAACAGGCATTGGTTTCCGAAT |  | 10538-10561 | 60 |  |
| OlVO | OlVO-HSP-F | GAACTGGGAGTGTTTATTTACAACC |  | 10229-10253 | 55 | 1302 |
|  | OlVO-HSP-R | CACTATAACATCCTTAACCGAGCT |  | 11507-11530 | 55 |  |
| OlVP | OlVP-HSP-F | GAATACTGTACCTTCACGAGAAGCT |  | 10604-10628 | 57 | 1024 |
|  | OlVP-HSP-R | AAACAGCATCCGGTAGAAACGA |  | 11606-11627 | 58 |  |
| OLYaV | OLYaV-F | GAACTACGTTTCACTTTGTGTGTACT |  | 10462-10487 | 56 | 736 |
|  | OLYaV-R | TCRAACATRGANGCAAAGAGAGC |  | 11175-11197 | 54 |  |
| OlVM | OlVM-CP-F | TCCCAAGATGTGTCCTACCTTCAG | CP | 6168-6191 | 55 | 954 |
|  | OlVM-CP-R | GGACGTGTTTTCCTTCACGTATCGT |  | 7098-7122 | 55 |  |
| OlVS | OlVS-F | TTATTCCGCTATGGTGGCTCTC | ORF2a | 677-698 | 60 | 671 |
|  | OlVS-R | AACCCATTCCTCAGTCTCACAC |  | 1326-1347 | 60 |  |
| OlVA  (22-0046) | OlVA-GSP1 | GCTTCTTGTGACAGAGA | 5’ terminal | 413-429 | 50 | - |
|  | OlVA-GSP2 | ATCTCCTCGAGATCGGGCAACCT | 5’ terminal | 248-270 | 62 | 270 |
|  | OlVA-GSP | GATTCGGCAAGTTGCGAATAGCAA | 3’ terminal | 20707-20730 | 60 | 380 |
| OlVV  (22-0046) | OlVV-GSP1 | AGAAGACTGTGGAGGTA | 5’ terminal | 572-588 | 51 | - |
|  | OlVV-GSP2 | ATGTCTACGGGGAAAGATGCGAAT | 5’ terminal | 313-336 | 62 | 336 |
|  | OlVV-GSP | GAAACTTCCAACACTCGCAAAGAG | 3’ terminal | 16328-16351 | 58 | 708 |
| OlVO  (22-0039) | OlVO-GSP1 | ACAGAACGGCTAGTAGT | 5’ terminal | 765-781 | 53 | - |
|  | OlVO-GSP2 | GCTGCCTTCTTGCGCTCATTAGC | 5’ terminal | 297-319 | 62 | 319 |
|  | OlVO-GSP | CAAATCAAAGAGAATCCCTCGAGTCA | 3’ terminal | 15577-15602 | 60 | 935 |
| OlVP  (22-0053) | OlVP-GSP1 | CTAAAGGAACCGAACTGGA | 5’ terminal | 448-466 | 42 | - |
|  | OlVP-GSP2 | ACCTCCTTCAATGCGGTCTCAGA | 5’ terminal | 378-400 | 60 | 400 |
|  | OlVP-GSP | CCAAGTACTGTGCGGAGAGAGTA | 3’ terminal | 15945-15967 | 60 | 645 |
| OlVM  (22-0051) | OlVM-GSP1 | CTTCTGGATCACTGATAAAGG | 5’ terminal | - | 53 | - |
|  | OlVM-GSP2 | GGCGACGCAGGATTTGGATTGG | 5’ terminal | 365-386 | 59 | 386 |
|  | OlVM-GSP | GACTACTTCGGTGGTCGGTTCA | 3’ terminal | 6819-6840 | 61 | 405 |
| OlVS  (22-0040) | OlVS-GSP1 | AGTCGATCACTTGGTCCAA | 5’ terminal | 501-519 | 54 | - |
|  | OlVS-GSP2 | CACGATAAGCACTTGAATCCGTACT | 5’ terminal | 318-342 | 58 | 342 |
|  | OlVS-GSP | CCTTGGTATGTATACCGTGTAGCT | 3’ terminal | 3847-3870 | 57 | 318 |

Supplementary Table S2: Primers designed in this study and used in PCR, RT-PCR and RACE assays with their associated genomic target region, melting temperature (Tm) and product size (RT-PCR). The accession numbers in the virus column represent the specific samples on which RACE was performed. Key: CP – Coat protein; Pol – Polyprotein; HSP70 – heat shock protein 70 homolog; OlAV – olive virus A OlVV – olive virus V; OlVO – olive virus O; OlVP – olive virus P; OLYaV – olive leaf yellowing-associated virus; OlVM – olive virus M; OlVS – olive virus S; GSP – Gene specific primer; -F – Forward; -R – Reverse.
