# Supplementary material for: Genomic characterization of novel viruses associated with Olea europaea L. in South Africa
Source: Arch Virol. 2024 Sep 27;169(10):210. doi: 10.1007/s00705-024-06132-1 (PMC11427506; doi:10.1007/s00705-024-06132-1)
Supplement: Supplementary file 6 — Supplementary Material 6 [file 705_2024_6132_MOESM6_ESM.docx]

| Virus/sample  **Supplementary Table S3**: Genome characters of novel viruses detected in olive tissue, including genome length in nucleotides (nt), open reading frame (ORF) putative gene location, product size in amino acids (aa), putative product and function, average amino acid sequence identity shared with the most closely related viral variant, molecular weight (MW) in kilodaltons (kDa) and isoelectric point (pI) for each open reading frame (ORF). **OlVA –** olive virus A; **OlVV** – olive virus V; **OlVO** – olive virus O; **OlVP** – olive virus P; **OlVS –** olive virus S; **OlVM** – olive virus M; | Length (nt) | ORF | Location (nt) | Putative product | Product size (aa) | Putative function | % aa identity | MW (kDa) | pI |
| --- | --- | --- | --- | --- | --- | --- | --- | --- | --- |
| OlVA/22-0040 | 21,087 | ORF1a | 211-9,552 | Polyprotein | 3,113 | Protease/methyltransferase/helicase | 39.6^1^ | 350.5 | 9.0 |
|  |  | ORF1b | 9,545-11,110 | RdRp | 521 | RNA-dependent RNA polymerase | 57.3^1^ | 60.1 | 6.2 |
|  |  | ORF2 | 11,377-12,075 | p33 | 232 | Thaumatin-like protein | 39.4^2^ | 26.2 | 4.5 |
|  |  | ORF3 | 12,319-13,971 | HSP70 | 550 | Heat shock protein 70 homolog | 45^2^ | 70.0 | 5.8 |
|  |  | ORF4 | 13,968-15,464 | HSP90 | 498 | Heat shock protein 90 homolog | 23.3^3^ | 58.5 | 9.0 |
|  |  | ORF5 | 15,573-16,457 | CP | 294 | Major coat protein | 38.1^4^ | 32.8 | 6.1 |
|  |  | ORF6 | 16,519-18,192 | mCP | 557 | Minor coat protein | 34.3^4^ | 64.0 | 8.7 |
|  |  | ORF7 | 18,235-19,254 | p39 | 339 | Putative 39kDa protein | n/a | 38.6 | 6.5 |
|  |  | ORF8 | 19,127-19,771 | p24 | 214 | Putative 24kDa protein | n/a | 24.2 | 5.9 |
|  |  | ORF9 | 19,771-20,091 | p17 | 106 | Putative 17kDa protein | n/a | 13.0 | 9.4 |
|  |  | ORF10 | 20,132-20,824 | p26 | 230 | Putative 26kDa protein | n/a | 26.2 | 7.7 |
| OlVV/22-0053 | 17,036 | ORF1a | 53-7,696 | Polyprotein | 2,547 | Protease/methyltransferase/helicase | 30.0^5^ | 290.3 | 9.1 |
|  |  | ORF1b | 7,650-9,212 | RdRp | 520 | RNA-dependent RNA polymerase | 62.6^6^ | 60.8 | 6.0 |
|  |  | ORF2 | 9,377-11,026 | HSP70 | 549 | Heat shock protein 70 homolog | 49.6^6^ | 62.4 | 5.8 |
|  |  | ORF3 | 10,956-11,216 | p10 | 86 | Putative 10kDa protein | n/a | 10.3 | 4.4 |
|  |  | ORF4 | 11,204-12,766 | HSP90 | 520 | Heat shock protein 90 homolog | 36.0^7^ | 60.8 | 8.2 |
|  |  | ORF5 | 12,768-13,694 | CP | 294 | Major coat protein | 36.0^7^ | 33.8 | 8.7 |
|  |  | ORF6 | 13,705-15,582 | mCP | 625 | Minor coat protein | 22.2^7^ | 73.5 | 6.1 |
|  |  | ORF7 | 15,579-16,250 | p26 | 223 | Putative 26kDa protein | n/a | 26.8 | 5.8 |
|  |  | ORF8 | 16,301-16,870 | p23 | 189 | Putative 23kDa protein | n/a | 22.6 | 7.0 |
| OlVO/22-0050 | 16,510 | ORF1a | 111-8,204 | Polyprotein | 2,697 | Protease/methyltransferase/helicase | 46.1^8^ | 304.7 | 9.0 |
|  |  | ORF1b | 7,942-9,624 | RdRp | 560 | RNA-dependent RNA polymerase | 73.6^8^ | 65.2 | 9.3 |
|  |  | ORF2 | 9,706-10,311 | p21 | 201 | Putative thaumatin-like protein | 62.6^9^ | 23.0 | 4.5 |
|  |  | ORF3 | 10,496-12,295 | HSP70 | 599 | Heat shock protein 70 homolog | 60.2^8^ | 66.9 | 6.4 |
|  |  | ORF4 | 12,156-13,718 | HSP90 | 520 | Heat shock protein 90 homolog | 56.8^8^ | 61.1 | 9.0 |
|  |  | ORF5 | 13,839-14,543 | CP | 234 | Coat protein | 62.7^8^ | 26.5 | 7.7 |
|  |  | ORF6 | 14,566-15,075 | p17 | 169 | Hypothetical protein | 47.9^8^ | 19.2 | 4.8 |
|  |  | ORF7 | 15,059-15,361 | p8 | 100 | Hypothetical protein | n/a | 13.0 | 9.0 |
|  |  | ORF8 | 15,449-16,048 | p23 | 199 | Putative 23kDa protein | 55.3^9^ | 23.2 | 5.5 |
|  |  | ORF9 | 16045-16,299 | p10 | 84 | Putative 10kDa protein | 56.1^9^ | 9.5 | 8.8 |
| OlVP/22-0040 | 16,591 | ORF1a | 132-8,186 | Polyprotein | 2,684 | Protease/methyltransferase/helicase | 45.7^8^ | 305.3 | 9.0 |
|  |  | ORF1b | 8,140-9,612 | RdRp | 386 | RNA-dependent RNA polymerase | 77.1^9^ | 44.9 | 8.5 |
|  |  | ORF2 | 9,894-10,460 | p21 | 188 | Putative thaumatin-like protein | 64.5^8^ | 21.3 | 4.7 |
|  |  | ORF3 | 10,562-12,412 | HSP70 | 616 | Heat shock protein 70 homolog | 61.3^8^ | 68.9 | 7.5 |
|  |  | ORF4 | 12300-13,865 | HSP90 | 521 | Heat shock protein 90 homolog | 56.0^8^ | 61.9 | 7.6 |
|  |  | ORF5 | 13,931-14,623 | CP | 230 | Coat protein | 66.4^8^ | 26.1 | 7.8 |
|  |  | ORF6 | 14,654-15,154 | p17 | 169 | Hypothetical protein | 48.7^8^ | 19.0 | 4.6 |
|  |  | ORF7 | 15,114-15,425 | p8 | 103 | Hypothetical protein | n/a | 11.8 | 9.9 |
|  |  | ORF8 | 15,515-16,114 | p23 | 199 | Putative 23kDa protein | 55.3^9^ | 23.5 | 6.8 |
|  |  | ORF9 | 16,111-16,371 | p10 | 86 | Putative 10kDa protein | 58.8^8^ | 10.0 | 8.9 |
| OlVS/21-0135 | 4,165 | ORF1 | 89-544 | MP | 151 | Movement protein | n/a | 17.7 | 5.1 |
|  |  | ORF2a | 547-2,289 | P2a | 580 | Polyprotein P2a | 39.5^10^ | 64.1 | 6.8 |
|  |  | ORF2b | 1,872-3,500 | P2b | 542 | RNA-dependent RNA polymerase | 58.9^11^ | 61.8 | 5.9 |
|  |  | ORF3 | 3,244-4,029 | CP | 261 | Coat protein | 50.4^12^ | 28.1 | 10.6 |
| OlVM/22-0047 | 7,227 | ORF1 | 124-6,234 | Pol | 2,036 | Polyprotein | 68.4^13^ | 226.5 | 7.9 |
|  |  | GP2 | 6235-7068 | CP | 277 | Coat protein | 79.9^13^ | 29.5 | 8.6 |

^1^ MG925331 grapevine leafroll associated virus 1; ^2^ NC_055482 pistachio ampelovirus A; ^3^ MW365400 citrus associated ampelovirus 1; ^4^ MW365402 citrus associated ampelovirus 2; ^5^ OM471839 cordyline virus 1, ^6^NC_016436 grapevine leafroll-associated virus 7, ^7^ MT533601 agapanthus velarivirus, ^8^ OK569886 olive leaf yellowing-associated virus, ^9^ MT809205 olive leaf yellowing-associated virus, ^10^ NC_002568 sesbania mosaic virus, ^11^ KX599170 southern cowpea mosaic virus ^12^ sobemovirus sp., ^13^ NC_013920 olive latent virus 3. n/a – No significant similarity to viral homologs
