# Supplementary material for: Genomic characterization of novel viruses associated with Olea europaea L. in South Africa
Source: Arch Virol. 2024 Sep 27;169(10):210. doi: 10.1007/s00705-024-06132-1 (PMC11427506; doi:10.1007/s00705-024-06132-1)
Supplement: Supplementary file 7 — Supplementary Material 7 [file 705_2024_6132_MOESM7_ESM.docx]

| Virus name | NCBI GenBank accession number | Sample number | Genome length (nt) | Average coverage (x) |
| --- | --- | --- | --- | --- |
| Olive virus A | OQ863252 | 22-0040 | 21,087 | 91.4 |
|  | OQ863253 | 22-0046 | 21.057 | 98.4 |
|  | OR912096 | 22-0047 | 21.057 | 98.0 |
|  | OR912097 | 22-0052 | 21,087 | 231.4 |
| Olive virus O | OR266370 | 22-0038 | 16,512 | 130.4 |
|  | OR266373 | 22-0039 | 16,512 | 33.7 |
|  | OR266371 | 22-0050 | 16,510 | 22.9 |
|  | OR266369 | 22-0051 | 16,512 | 59.7 |
|  | OR266372 | 22-0054 | 16,507 | 11.6 |
| Olive virus P | OR270933 | 22-0040 | 16,590 | 45.8 |
|  | OR270934 | 22-0052 | 16,590 | 80.6 |
|  | OR270932 | 22-0053 | 16,590 | 14.8 |
| Olive virus V | OQ863255 | 22-0038 | 17,035 | 273.0 |
|  | OQ863256 | 22-0039 | 17,035 | 139.3 |
|  | OQ863257 | 22-0040 | 17,035 | 109.4 |
|  | OQ863258 | 22-0041 | 17,035 | 208.0 |
|  | OQ863259 | 22-0042 | 17,035 | 651.0 |
|  | OQ863260 | 22-0043 | 17,035 | 75.5 |
|  | OQ863261 | 22-0044 | 17,035 | 41.3 |
|  | OQ863262 | 22-0046 | 17,035 | 73.2 |
|  | OR912097 | 22-0047 | 17,035 | 73.0 |
|  | OQ863263 | 22-0049 | 17,035 | 426.3 |
|  | OQ863264 | 22-0051 | 17,035 | 145.1 |
|  | OQ863265 | 22-0052 | 17,035 | 315.0 |
|  | OQ863266 | 22-0053 | 17,036 | 126.9 |
|  | OR912098 | 22-0054 | 16,173^*^ | 56.9 |
| Olive leaf yellowing-associated virus | OR346819 | 22-0038 | 16,723 | 69.1 |
|  | OR346820 | 22-0038 | 16,671 | 76.3 |
|  | OR346821 | 22-0040 | 16,730 | 9.7 |
|  | OR346822 | 22-0041 | 16732 | 321.0 |
|  | OR346823 | 22-0042 | 16,129 | 131.0 |
|  | OR346824 | 22-0043 | 16,715 | 8.7 |
|  | OR346825 | 22-0045 | 16,283 | 32.3 |
|  | OR346826 | 22-0045 | 16,282 | 24.3 |
|  | OR346827 | 22-0045 | 16,840 | 101.7 |
|  | OR346828 | 22-0045 | 16,517 | 27.9 |
|  | OR346829 | 22-0048 | 16,738 | 25.9 |
|  | OR346830 | 22-0049 | 16,751 | 59.7 |
|  | OR346831 | 22-0050 | 16,839 | 22.6 |
|  | OR346832 | 22-0052 | 16,678 | 80.6 |
| Olive virus S | OR252871 | 22-0038 | 4,160 | 2,400.5 |
|  | OR252872 | 22-0040 | 4,165 | 65,477.6 |
|  | OR252873 | 22-0043 | 4,157 | 10,938.0 |
|  | OR252874 | 22-0044 | 4,164 | 7,110.3 |
|  | OR252875 | 22-0045 | 4,165 | 267,629.9 |
|  | OR252876 | 22-0046 | 4,165 | 21,575.8 |
|  | OR252877 | 22-0049 | 4,165 | 133,148.4 |
|  | OR252878 | 22-0051 | 4,165 | 58,707.6 |
|  | OR252879 | 22-0052 | 4,157 | 17,005.7 |
|  | OR252867 | 22-0053 | 4,165 | 15,632.7 |
| Olive virus M | OR912100 | 22-0047 | 7,224 | 6.6 |
|  | OR912101 | 22-0051 | 7,223 | 56.3 |

Supplementary Table S4: List of viruses from this study with their associated sample and NCBI GenBank accession numbers and source sample numbers. Genome length (in nucleotides (nt)), as well as the average coverage (in times coverage (x)) are also presented
